# Crystal structure of 4-[(adamantan-1-yl)amino]­naphthalene-1,2-dione

**DOI:** 10.1107/S2056989018017917

**Published:** 2019-01-01

**Authors:** Guy Lamoureux, Mónica Alvarado-Rojas, Leslie W. Pineda

**Affiliations:** a Escuela de Química, Universidad de Costa Rica, 2060, San José, Costa Rica; b Centro de investigación en Productos Naturales (CIPRONA), Universidad de Costa Rica, 2060, San José, Costa Rica; c Centro de Electroquímica y Energía Química (CELEQ), Universidad de Costa Rica, 2060, San José, Costa Rica

**Keywords:** crystal structure, adamantane, N—H⋯O hydrogen bonding, intra­molecular hydrogen bonding, naphtho­quinone

## Abstract

The title compound, C_20_H_21_NO_2_, an example of a stable 1,2-naphtho­quinone, was determined by single-crystal X-ray diffraction analysis at 100 K. This structure illustrates steric buttressing of the adamantanyl group, forcing the N—H group into the coplanar aromatic C—H. The presence of strong delocalization between the planar N atom at the 4-position and the carbonyl group at the 2-position is indicated. In the crystal, C—H⋯O and C—H⋯π inter­actions link the mol­ecules into a three-dimensional network.

## Abstract 2

The title compound, an example of a stable 1,2-naphtho­quinone, illustrates steric buttressing of the adamantanyl group.

## Chemical context   

The formation of 4-amino-1,2-naphtho­quinones is important in the colorimetric analysis (Folin analysis) of amines (Folin, 1922 ▸). However, the isolation and characterization of these amino­quinones is not common (Asahi *et al.*, 1984 ▸). In the literature, it is reported that the yields for the formation of 1,2-naphtho­quinones with a primary amino group in the 4-position are greatly inferior to those of secondary amino groups (Bullock *et al.*, 1970 ▸). These inferior yields may be due to the equilibrium of amine/imine tautomeric forms (Yano *et al.*, 1980 ▸; Fragoso *et al.*, 2010 ▸), which would complicate the identification of 4-primary amino-1,2-naphtho­quinones (Hartke & Lohmann, 1983 ▸). As part of our work on the synthesis and properties of naphtho­quinones (Lamoureux *et al.*, 2008 ▸), we were inter­ested to prepare and analyze the structure of the title compound 4-[(adamantan-1-yl)amino]­naphthalene-1,2-dione, also known as 4-(1-adamantanyl­amino)-1,2-naphtho­quinone). To the best of our knowledge, the hybrid of a naphtho­quinone core with an adamantanyl substituent is not known in the literature (Lamoureux & Artavia, 2010 ▸). 
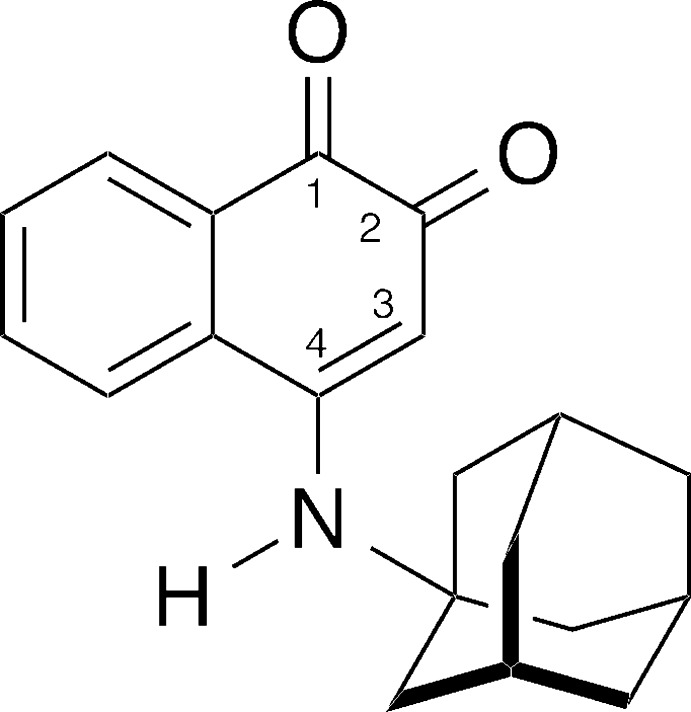


## Structural commentary   

**Figure 1:**
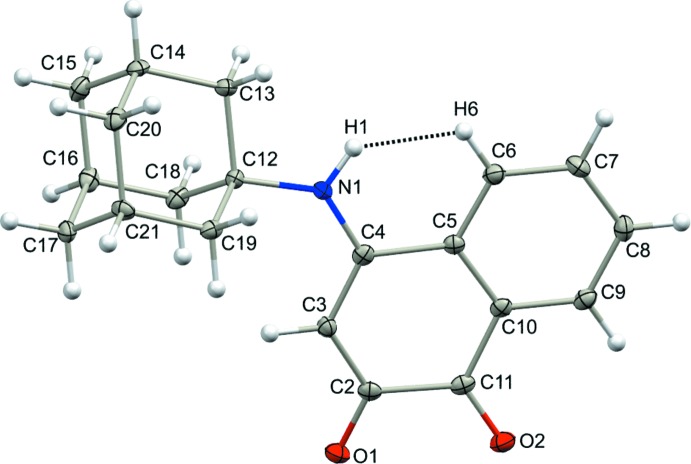
The mol­ecular structure of the title compound with displacement ellipsoids drawn at the 50% probability level. The steric compression is shown as a dotted line.

In the mol­ecule of the title compound (Fig. 1 ▸), the C=O bond length of the carbonyl group at the 1-position [C11=O2 = 1.216 (2) Å] is shorter than the other at the 2-position [C2=O1 = 1.241 (2) Å], suggesting strong delocalization from the trigonal-planar nitro­gen at the 4-position, causing a decrease of the double-bond character at the C2 carbonyl (vinyl­ogous amide), whereas the C1 carbonyl atom is unaffected. Further evidence of this delocalization is shown by a short N1—C4 bond distance [1.346 (2) Å], which is inter­mediate between the C—N and C=N bond distances observed in a related quinone amine/imine structure (Lamoureux *et al.*, 2018 ▸). The aliphatic bond distance [N1—C12 = 1.482 (2) Å] is longer than expected, but may be caused by the bulky adamantanyl group. Further evidence of the steric effect of the adamantanyl group is shown by the large angle at the planar nitro­gen atom [C4—N1—C12 = 131.1 (2)°] compared to the ideal value of 120°. Most strikingly, the compression on one side of the adamantane ring causes another through-space compression between the NH group and the aromatic ring of the naphtho­quinone ring system (H1⋯H6 = 1.82 Å; H1⋯C6 = 2.37 Å).

The fused quinone ring adopts a flattened envelope conformation, with atom C2 as the flap (displaced by 0.0687 (18) Å from the plane through the other atoms); the O1—C2—C11—O2 torsion angle formed by the two carbonyl groups is −6.1 (3)°. The C10—C11—C2 angle of 117.8 (2)°, C10—C11—C2 angle of 117.9 (2)° and C2—C3—C4 angle of 123.5 (2)° show the largest deviations from the ideal value of 120°. The aromatic ring is planar, as expected, and has inter­nal bond angles that range from 117.9 (2) to 120.9 (2)°.

## Supra­molecular features   

**Figure 2:**
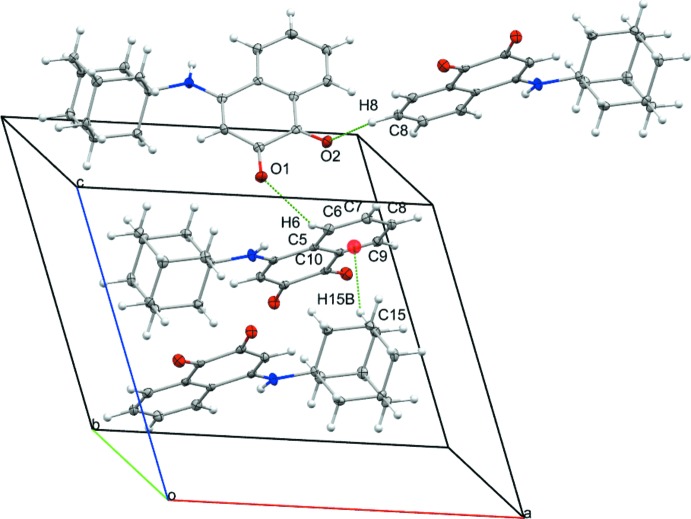
Partial crystal packing of the title compound. C—H⋯O inter­actions are shown as dashed lines. The C—H⋯*π* inter­action is shown as a green dashed line between the orange centroid of the aromatic ring and the hydrogen atom H15*B*.

In the crystal structure of the title compound (Fig. 2 ▸), mol­ecules are linked into a three-dimensional network by C—H⋯O hydrogen bonds (Table 1 ▸) involving as donors the C—H groups of both the adamantanyl system and the benzene ring. The crystal packing is further consolidated by C—H⋯π inter­actions. There are no π–π inter­actions, the aromatic rings being separated by more than 6 Å.

## Database survey   

A search of the Cambridge Structural Database (Version 5.39, update February 2018; Groom *et al.*, 2016 ▸) for the substructure 4-amino-1,2-naphtho­quinone yielded seven hits. However, only one structure (refcode ZARNOY; Hatfield *et al.*, 2017 ▸) contains a primary amine (aniline) in the 4-position. The distance between the N—H group and the coplanar aromatic hydrogen atom [1.93 (4) Å] in this structure is longer than in the title compound, probably due to the smaller size of the nitro­gen substituent. Surprisingly, the carbonyl groups in ZARNOY are almost coplanar [torsion angle of 0.2 (5)°]. In the same reference (Hatfield *et al.*, 2017 ▸), another structure is reported (refcode ZARPAM) with a secondary amine (*N*-methyl­aniline), which has a completely different structure from ZARNOY: the nitro­gen is not planar, the amino moiety is twisted with respect to the naphtho­quinone plane and the C4—N bond distance is greater in the case of the secondary amine. The authors summarize the differences between the structures and rationalize these differences using the concept of tautomerization (more accurately greater delocalization) in the structure with the primary amine.

Of the other structures in the database, four structures contain a secondary amine connected at the 4-position. Two structures (refcodes DMANPQ10 and EANAPQ10; Bechtel *et al.*, 1976 ▸), involve the simple aliphatic amines di­methyl­amine and di­ethyl­amine. One structure (SEJZIQ; Ukhin *et al.*, 1997 ▸) combines the cyclic morpholine with 1,2-naphtho­quinone. The structure of XANRUB (Singh *et al.*, 2011 ▸) contains a carbazole moiety at the 4-position of the 1,2-naphtho­quinone unit.

Finally, one structure AMNPQH10 (Aime *et al.*, 1970 ▸) is anomalous since it contains an –NH_2_ group at the 4-position, yet has bond and angle parameters completely different from the other mol­ecules. Based on our analysis, this structure from 1970 should be re-analyzed to determine whether it could be best refined as an imino­quinone.

## Synthesis and crystallization   

The synthesis of 4-[(adamantan-1-yl)amino]­naphthalene-1,2-dione is based on a new procedure (complete publication in progress). In a reaction tube were mixed 740 mg (2.00 mmol) of 1,2-naphtho­quinone-4-sulfonic acid cesium salt, 76 mg (0.50 mmol, 1 equiv) of adamantan-1-amine, and 302 mg (1.00 mmol, 2 equiv) of tetra­butyl­ammonium acetate. The solids were dissolved in *tert*-amyl alcohol (5.0 mL). A cellulose extraction thimble with Li_2_CO_3_ was placed above the reaction mixture. This solution was stirred at 393 K under a nitro­gen atmosphere for 5 h. After being allowed to cool to room temperature, the dark-brown solution was diluted with toluene (30 mL), filtered and concentrated under reduced pressure. A brownish-red solid (503 mg) of the crude product was obtained. The crude product was further purified by column chromatography using silica gel with a gradient solvent elution [100% di­chloro­methane (CH_2_Cl_2_) and then di­chloro­methane/2-propanol (CH_2_Cl_2_/C_3_H_8_O, 9:1 *v*/*v*)]; the fractions were dried under vacuum to yield 72 mg of a dark-orange solid product (47% yield), determined pure by NMR analysis. Part of the purified product was re-dissolved in heptane and cooled to 203 K for crystallization. Red crystalline blocks suitable for X-ray analysis were obtained, m.p. 522 K (decomposition) determined using a Fisher–Johns melting-point apparatus with calibrated thermometer. ^1^H NMR (600 MHz, CDCl_3_) δ 8.20–8.21 (*d*, *J* = 7.6 Hz, 1 H), 7.66–7.69 (*t*, *J* = 7.8 Hz, 1 H), 7.58–7.61 (*t*, *J* = 7.6 Hz, 1 H), 7.39–7.40 (*d*, *J* = 7.9 Hz, 1 H), 6.20 (*br s*, 1H), 5.47 (*br s*, 1 H), 2.22 (*br s*, 3 H), 2.16 (*br s*, 6 H), 1.73–1.79 (*m*, 6 H).

## Refinement   

Crystal data, data collection and structure refinement details are summarized in Table 2 ▸. The N-bound H atom was located in a difference-Fourier map and refined as riding, with N—H = 0.88 Å, and with *U*_iso_(H) = 1.2 *U*_eq_(N). All other H atoms were placed geometrically and refined using a riding-atom approximation, with C—H = 0.95–1.00 Å, and with *U*_iso_(H) = 1.2*U*_eq_(C).

## Supplementary Material

Crystal structure: contains datablock(s) global, I. DOI: 10.1107/S2056989018017917/rz5250sup1.cif

Structure factors: contains datablock(s) I. DOI: 10.1107/S2056989018017917/rz5250Isup2.hkl

Supporting information file. DOI: 10.1107/S2056989018017917/rz5250Isup3.cml

CCDC reference: 1876987

Additional supporting information:  crystallographic information; 3D view; checkCIF report

## Figures and Tables

**Table 1:** Hydrogen-bond geometry (Å, °) *Cg*1 is the centroid of the C5–C10 ring.

*D*—H⋯*A*	*D*—H	H⋯*A*	*D*⋯*A*	*D*—H⋯*A*
C6—H6⋯O1^i^	0.95	2.59	3.385 (3)	142
C8—H8⋯O2^ii^	0.95	2.47	3.231 (2)	137
C13—H13*A*⋯O1^i^	0.99	2.51	3.400 (2)	150
C15—H15*B*⋯*Cg*1^iii^	0.99	2.74	3.587 (2)	144

Symmetry codes: (i) 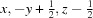; (ii) 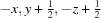; (iii) 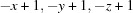.

**Table 2:** Experimental details

Crystal data	
Chemical formula	C_20_H_21_NO_2_
*M*_r_	307.38
Crystal system, space group	Monoclinic, *P*2_1_/*c*
Temperature (K)	100
*a*, *b*, *c* (Å)	12.8487 (5), 10.8187 (4), 11.8469 (5)
β (°)	112.248 (1)
*V* (Å^3^)	1524.20 (10)
*Z*	4
Radiation type	Mo *K*α
μ (mm^−1^)	0.09
Crystal size (mm)	0.20 × 0.15 × 0.10

Data collection	
Diffractometer	Bruker D8 Venture
Absorption correction	Multi-scan (*SADABS*; Bruker, 2015 ▸)
*T*_min_, *T*_max_	0.702, 0.746
No. of measured, independent and observed [*I* > 2σ(*I*)] reflections	31552, 3494, 2412
*R*_int_	0.075
(sin θ/λ)_max_ (Å^−1^)	0.650

Refinement	
*R*[*F* ^2^ > 2σ(*F* ^2^)], *wR*(*F* ^2^), *S*	0.056, 0.121, 1.05
No. of reflections	3494
No. of parameters	208
H-atom treatment	H-atom parameters constrained
Δρ_max_, Δρ_min_ (e Å^−3^)	0.36, −0.32

Computer programs: *APEX3* and *SAINT* (Bruker, 2015 ▸), *SHELXT* (Sheldrick, 2015*a* ▸), *SHELXL* (Sheldrick, 2015*b* ▸) and *shelXle* (Hübschle *et al.*, 2011 ▸).

## supplementary crystallographic information

### Crystal data

**Table:**

C_20_H_21_NO_2_	*F*(000) = 656
*M**_r_* = 307.38	*D*_x_ = 1.339 Mg m^−^^3^
Monoclinic, *P*2_1_/*c*	Mo *K*α radiation, λ = 0.71073 Å
*a* = 12.8487 (5) Å	Cell parameters from 80 reflections
*b* = 10.8187 (4) Å	θ = 3.5–20.0°
*c* = 11.8469 (5) Å	µ = 0.09 mm^−^^1^
β = 112.248 (1)°	*T* = 100 K
*V* = 1524.20 (10) Å^3^	Block, translucent intense orange-red
*Z* = 4	0.20 × 0.15 × 0.10 mm

### Data collection

**Table:**

Bruker D8 Venture diffractometer	3494 independent reflections
Radiation source: Incoatec Microsource	2412 reflections with *I* > 2σ(*I*)
Mirrors monochromator	*R*_int_ = 0.075
Detector resolution: 10.4167 pixels mm^-1^	θ_max_ = 27.5°, θ_min_ = 2.5°
ω scans	*h* = −16→16
Absorption correction: multi-scan (SADABS; Bruker, 2015)	*k* = −14→14
*T*_min_ = 0.702, *T*_max_ = 0.746	*l* = −15→15
31552 measured reflections	

### Refinement

**Table:**

Refinement on *F*^2^	Primary atom site location: structure-invariant direct methods
Least-squares matrix: full	Secondary atom site location: difference Fourier map
*R*[*F*^2^ > 2σ(*F*^2^)] = 0.056	Hydrogen site location: inferred from neighbouring sites
*wR*(*F*^2^) = 0.121	H-atom parameters constrained
*S* = 1.05	*w* = 1/[σ^2^(*F*_o_^2^) + (0.0492*P*)^2^ + 0.826*P*] where *P* = (*F*_o_^2^ + 2*F*_c_^2^)/3
3494 reflections	(Δ/σ)_max_ < 0.001
208 parameters	Δρ_max_ = 0.36 e Å^−^^3^
0 restraints	Δρ_min_ = −0.31 e Å^−^^3^

### Special details

**Table:**

Geometry. All esds (except the esd in the dihedral angle between two l.s. planes) are estimated using the full covariance matrix. The cell esds are taken into account individually in the estimation of esds in distances, angles and torsion angles; correlations between esds in cell parameters are only used when they are defined by crystal symmetry. An approximate (isotropic) treatment of cell esds is used for estimating esds involving l.s. planes.

### Fractional atomic coordinates and isotropic or equivalent isotropic displacement parameters (Å^2^)

**Table:**

	*x*	*y*	*z*	*U*_iso_*/*U*_eq_	
O1	0.38228 (11)	0.03240 (12)	0.55184 (12)	0.0167 (3)	
O2	0.15850 (11)	0.07574 (12)	0.43684 (12)	0.0174 (3)	
N1	0.45787 (13)	0.36387 (14)	0.33029 (14)	0.0130 (3)	
H1	0.4241	0.4268	0.2837	0.016*	
C2	0.35129 (15)	0.11642 (16)	0.47491 (16)	0.0115 (4)	
C3	0.42405 (15)	0.18810 (17)	0.43727 (16)	0.0119 (4)	
H3	0.5014	0.1664	0.4668	0.014*	
C4	0.38867 (15)	0.28844 (16)	0.35958 (16)	0.0106 (4)	
C5	0.26636 (15)	0.32093 (16)	0.30318 (16)	0.0099 (4)	
C6	0.22581 (16)	0.41901 (17)	0.22207 (17)	0.0136 (4)	
H6	0.2768	0.4671	0.1996	0.016*	
C7	0.11207 (16)	0.44738 (17)	0.17363 (17)	0.0145 (4)	
H7	0.0861	0.5149	0.1188	0.017*	
C8	0.03608 (16)	0.37845 (18)	0.20441 (17)	0.0148 (4)	
H8	−0.0416	0.3991	0.172	0.018*	
C9	0.07435 (16)	0.27905 (17)	0.28295 (17)	0.0135 (4)	
H9	0.0224	0.2304	0.3034	0.016*	
C10	0.18809 (15)	0.24991 (16)	0.33209 (16)	0.0107 (4)	
C11	0.22506 (16)	0.14264 (16)	0.41573 (16)	0.0114 (4)	
C12	0.58113 (15)	0.35926 (16)	0.36293 (16)	0.0105 (4)	
C13	0.61093 (15)	0.48247 (17)	0.31748 (17)	0.0131 (4)	
H13A	0.5659	0.4918	0.2292	0.016*	
H13B	0.5922	0.552	0.3608	0.016*	
C14	0.73627 (15)	0.48667 (17)	0.33974 (17)	0.0133 (4)	
H14	0.7542	0.567	0.3093	0.016*	
C15	0.80594 (16)	0.47438 (18)	0.47649 (17)	0.0156 (4)	
H15A	0.8871	0.476	0.4912	0.019*	
H15B	0.7896	0.5444	0.521	0.019*	
C16	0.77630 (16)	0.35233 (18)	0.52241 (17)	0.0140 (4)	
H16	0.8215	0.3441	0.6118	0.017*	
C17	0.80384 (16)	0.24419 (18)	0.45448 (17)	0.0145 (4)	
H17A	0.8852	0.2441	0.4703	0.017*	
H17B	0.7851	0.165	0.4842	0.017*	
C18	0.65032 (15)	0.35023 (18)	0.50031 (16)	0.0133 (4)	
H18A	0.6318	0.2726	0.5329	0.016*	
H18B	0.6321	0.4206	0.543	0.016*	
C19	0.61046 (15)	0.25234 (17)	0.29482 (16)	0.0118 (4)	
H19A	0.5923	0.1724	0.3238	0.014*	
H19B	0.5655	0.2592	0.2063	0.014*	
C20	0.76438 (16)	0.37983 (17)	0.27165 (17)	0.0144 (4)	
H20A	0.8453	0.382	0.2853	0.017*	
H20B	0.7205	0.388	0.183	0.017*	
C21	0.73612 (15)	0.25706 (17)	0.31772 (17)	0.0128 (4)	
H21	0.7551	0.1872	0.2736	0.015*	

### Atomic displacement parameters (Å^2^)

**Table:**

	*U*^11^	*U*^22^	*U*^33^	*U*^12^	*U*^13^	*U*^23^
O1	0.0177 (7)	0.0131 (7)	0.0193 (7)	0.0022 (6)	0.0070 (6)	0.0057 (6)
O2	0.0172 (7)	0.0148 (7)	0.0198 (8)	−0.0043 (6)	0.0068 (6)	0.0024 (6)
N1	0.0103 (8)	0.0108 (8)	0.0171 (8)	0.0015 (6)	0.0042 (7)	0.0050 (6)
C2	0.0149 (10)	0.0087 (9)	0.0118 (9)	0.0001 (7)	0.0059 (8)	−0.0028 (7)
C3	0.0101 (10)	0.0113 (9)	0.0140 (10)	0.0008 (8)	0.0041 (8)	−0.0008 (8)
C4	0.0123 (10)	0.0103 (9)	0.0103 (9)	−0.0008 (7)	0.0055 (8)	−0.0043 (7)
C5	0.0121 (9)	0.0091 (9)	0.0091 (9)	−0.0001 (7)	0.0046 (8)	−0.0027 (7)
C6	0.0144 (10)	0.0111 (9)	0.0159 (10)	−0.0012 (8)	0.0065 (8)	−0.0005 (8)
C7	0.0165 (10)	0.0122 (10)	0.0140 (10)	0.0032 (8)	0.0048 (8)	0.0029 (8)
C8	0.0108 (9)	0.0174 (10)	0.0154 (10)	0.0017 (8)	0.0040 (8)	−0.0020 (8)
C9	0.0146 (10)	0.0121 (9)	0.0154 (10)	−0.0025 (8)	0.0076 (8)	−0.0028 (8)
C10	0.0125 (9)	0.0091 (9)	0.0109 (9)	−0.0012 (7)	0.0050 (8)	−0.0038 (7)
C11	0.0157 (10)	0.0103 (9)	0.0091 (9)	−0.0028 (8)	0.0058 (8)	−0.0038 (7)
C12	0.0088 (9)	0.0100 (9)	0.0121 (9)	−0.0004 (7)	0.0034 (7)	0.0003 (7)
C13	0.0137 (10)	0.0084 (9)	0.0172 (10)	−0.0003 (7)	0.0060 (8)	0.0022 (7)
C14	0.0144 (10)	0.0092 (9)	0.0177 (10)	−0.0019 (7)	0.0075 (8)	0.0028 (8)
C15	0.0124 (10)	0.0166 (10)	0.0171 (10)	−0.0040 (8)	0.0050 (8)	−0.0043 (8)
C16	0.0126 (10)	0.0188 (10)	0.0093 (9)	−0.0014 (8)	0.0027 (8)	0.0010 (8)
C17	0.0100 (9)	0.0141 (10)	0.0200 (10)	0.0021 (8)	0.0064 (8)	0.0042 (8)
C18	0.0139 (10)	0.0156 (10)	0.0111 (9)	−0.0027 (8)	0.0055 (8)	−0.0007 (8)
C19	0.0141 (10)	0.0109 (9)	0.0102 (9)	−0.0027 (8)	0.0042 (8)	−0.0003 (7)
C20	0.0141 (10)	0.0169 (10)	0.0139 (10)	−0.0012 (8)	0.0073 (8)	0.0014 (8)
C21	0.0150 (10)	0.0099 (9)	0.0151 (10)	0.0024 (8)	0.0075 (8)	−0.0008 (7)

### Geometric parameters (Å, º)

**Table:**

O1—C2	1.241 (2)	C13—C14	1.531 (3)
O2—C11	1.216 (2)	C13—H13A	0.99
N1—C4	1.346 (2)	C13—H13B	0.99
N1—C12	1.482 (2)	C14—C20	1.529 (3)
N1—H1	0.88	C14—C15	1.531 (3)
C2—C3	1.411 (3)	C14—H14	1.0
C2—C11	1.530 (3)	C15—C16	1.530 (3)
C3—C4	1.384 (3)	C15—H15A	0.99
C3—H3	0.95	C15—H15B	0.99
C4—C5	1.498 (3)	C16—C17	1.535 (3)
C5—C6	1.393 (3)	C16—C18	1.539 (3)
C5—C10	1.407 (2)	C16—H16	1.0
C6—C7	1.387 (3)	C17—C21	1.528 (3)
C6—H6	0.95	C17—H17A	0.99
C7—C8	1.383 (3)	C17—H17B	0.99
C7—H7	0.95	C18—H18A	0.99
C8—C9	1.385 (3)	C18—H18B	0.99
C8—H8	0.95	C19—C21	1.533 (2)
C9—C10	1.389 (3)	C19—H19A	0.99
C9—H9	0.95	C19—H19B	0.99
C10—C11	1.482 (3)	C20—C21	1.531 (3)
C12—C18	1.534 (3)	C20—H20A	0.99
C12—C19	1.537 (2)	C20—H20B	0.99
C12—C13	1.539 (2)	C21—H21	1.0
			
C4—N1—C12	131.11 (16)	C13—C14—C15	109.58 (15)
C4—N1—H1	114.4	C20—C14—H14	109.5
C12—N1—H1	114.4	C13—C14—H14	109.5
O1—C2—C3	124.61 (17)	C15—C14—H14	109.5
O1—C2—C11	117.51 (16)	C16—C15—C14	109.06 (15)
C3—C2—C11	117.87 (16)	C16—C15—H15A	109.9
C4—C3—C2	123.46 (17)	C14—C15—H15A	109.9
C4—C3—H3	118.3	C16—C15—H15B	109.9
C2—C3—H3	118.3	C14—C15—H15B	109.9
N1—C4—C3	124.37 (17)	H15A—C15—H15B	108.3
N1—C4—C5	115.26 (16)	C15—C16—C17	109.47 (15)
C3—C4—C5	120.37 (16)	C15—C16—C18	109.85 (16)
C6—C5—C10	117.95 (17)	C17—C16—C18	109.63 (15)
C6—C5—C4	122.78 (16)	C15—C16—H16	109.3
C10—C5—C4	119.27 (16)	C17—C16—H16	109.3
C7—C6—C5	120.93 (17)	C18—C16—H16	109.3
C7—C6—H6	119.5	C21—C17—C16	109.55 (15)
C5—C6—H6	119.5	C21—C17—H17A	109.8
C8—C7—C6	120.67 (18)	C16—C17—H17A	109.8
C8—C7—H7	119.7	C21—C17—H17B	109.8
C6—C7—H7	119.7	C16—C17—H17B	109.8
C7—C8—C9	119.31 (18)	H17A—C17—H17B	108.2
C7—C8—H8	120.3	C12—C18—C16	109.22 (14)
C9—C8—H8	120.3	C12—C18—H18A	109.8
C8—C9—C10	120.49 (17)	C16—C18—H18A	109.8
C8—C9—H9	119.8	C12—C18—H18B	109.8
C10—C9—H9	119.8	C16—C18—H18B	109.8
C9—C10—C5	120.63 (17)	H18A—C18—H18B	108.3
C9—C10—C11	118.54 (16)	C21—C19—C12	109.46 (14)
C5—C10—C11	120.83 (17)	C21—C19—H19A	109.8
O2—C11—C10	122.06 (17)	C12—C19—H19A	109.8
O2—C11—C2	120.08 (16)	C21—C19—H19B	109.8
C10—C11—C2	117.85 (15)	C12—C19—H19B	109.8
N1—C12—C18	114.32 (15)	H19A—C19—H19B	108.2
N1—C12—C19	109.79 (14)	C14—C20—C21	109.42 (14)
C18—C12—C19	110.51 (15)	C14—C20—H20A	109.8
N1—C12—C13	105.21 (14)	C21—C20—H20A	109.8
C18—C12—C13	107.78 (15)	C14—C20—H20B	109.8
C19—C12—C13	108.98 (14)	C21—C20—H20B	109.8
C14—C13—C12	110.50 (15)	H20A—C20—H20B	108.2
C14—C13—H13A	109.5	C17—C21—C20	109.90 (15)
C12—C13—H13A	109.5	C17—C21—C19	108.87 (14)
C14—C13—H13B	109.5	C20—C21—C19	109.98 (15)
C12—C13—H13B	109.5	C17—C21—H21	109.4
H13A—C13—H13B	108.1	C20—C21—H21	109.4
C20—C14—C13	109.26 (15)	C19—C21—H21	109.4
C20—C14—C15	109.40 (15)		
			
O1—C2—C3—C4	−173.98 (18)	C4—N1—C12—C19	71.4 (2)
C11—C2—C3—C4	6.9 (3)	C4—N1—C12—C13	−171.49 (17)
C12—N1—C4—C3	3.9 (3)	N1—C12—C13—C14	−176.98 (14)
C12—N1—C4—C5	−177.13 (16)	C18—C12—C13—C14	60.65 (19)
C2—C3—C4—N1	174.46 (17)	C19—C12—C13—C14	−59.31 (19)
C2—C3—C4—C5	−4.5 (3)	C12—C13—C14—C20	59.72 (19)
N1—C4—C5—C6	2.6 (2)	C12—C13—C14—C15	−60.13 (19)
C3—C4—C5—C6	−178.38 (17)	C20—C14—C15—C16	−60.95 (19)
N1—C4—C5—C10	−177.72 (15)	C13—C14—C15—C16	58.82 (19)
C3—C4—C5—C10	1.3 (2)	C14—C15—C16—C17	60.51 (19)
C10—C5—C6—C7	1.5 (3)	C14—C15—C16—C18	−59.93 (19)
C4—C5—C6—C7	−178.80 (17)	C15—C16—C17—C21	−59.66 (19)
C5—C6—C7—C8	−0.3 (3)	C18—C16—C17—C21	60.90 (19)
C6—C7—C8—C9	−1.0 (3)	N1—C12—C18—C16	−177.36 (15)
C7—C8—C9—C10	1.1 (3)	C19—C12—C18—C16	58.19 (19)
C8—C9—C10—C5	0.1 (3)	C13—C12—C18—C16	−60.80 (19)
C8—C9—C10—C11	−179.99 (17)	C15—C16—C18—C12	61.65 (19)
C6—C5—C10—C9	−1.4 (3)	C17—C16—C18—C12	−58.69 (19)
C4—C5—C10—C9	178.86 (16)	N1—C12—C19—C21	173.69 (14)
C6—C5—C10—C11	178.73 (16)	C18—C12—C19—C21	−59.31 (19)
C4—C5—C10—C11	−1.0 (2)	C13—C12—C19—C21	58.95 (18)
C9—C10—C11—O2	4.3 (3)	C13—C14—C20—C21	−59.63 (19)
C5—C10—C11—O2	−175.85 (16)	C15—C14—C20—C21	60.33 (19)
C9—C10—C11—C2	−176.47 (16)	C16—C17—C21—C20	59.08 (19)
C5—C10—C11—C2	3.4 (2)	C16—C17—C21—C19	−61.43 (19)
O1—C2—C11—O2	−6.1 (3)	C14—C20—C21—C17	−59.49 (19)
C3—C2—C11—O2	173.12 (16)	C14—C20—C21—C19	60.35 (19)
O1—C2—C11—C10	174.65 (16)	C12—C19—C21—C17	60.33 (19)
C3—C2—C11—C10	−6.1 (2)	C12—C19—C21—C20	−60.13 (18)
C4—N1—C12—C18	−53.4 (3)		

### Hydrogen-bond geometry (Å, º)

Cg1 is the centroid of the C5–C10 ring.

**Table:**

*D*—H···*A*	*D*—H	H···*A*	*D*···*A*	*D*—H···*A*
C6—H6···O1^i^	0.95	2.59	3.385 (3)	142
C8—H8···O2^ii^	0.95	2.47	3.231 (2)	137
C13—H13*A*···O1^i^	0.99	2.51	3.400 (2)	150
C15—H15*B*···*Cg*1^iii^	0.99	2.74	3.587 (2)	144

Symmetry codes: (i) *x*, −*y*+1/2, *z*−1/2; (ii) −*x*, *y*+1/2, −*z*+1/2; (iii) −*x*+1, −*y*+1, −*z*+1.
